# Development and evaluation of an ovarian cancer prognostic model based on adaptive immune-related genes

**DOI:** 10.1097/MD.0000000000042030

**Published:** 2025-04-04

**Authors:** Huangmin Shi, Lijuan Li, Linying Zhou, Caiping Hong

**Affiliations:** a Department of Obstetrics and Gynaecology, People’s Hospital of Shangcheng District, Hangzhou, China; b Health Management Center, Longquan People’s Hospital, Longquan, Zhejiang, China; c Department of Obstetrics and Gynaecology, Longquan People’s Hospital, Longquan, Zhejiang, China.

**Keywords:** adaptive immune, enrichment analysis, immune infiltration, nomogram, ovarian cancer

## Abstract

The adaptive immune system plays a vital role in cancer prevention and control. However, research investigating the predictive value of adaptive immune-related genes (AIRGs) in ovarian cancer (OC) prognosis is limited. This study aims to explore the functional roles of AIRGs in OC. Transcriptomic, clinical-pathological, and prognostic data for OC were downloaded from public databases. Differential expression analysis, univariate, and Lasso Cox regression analyses were utilized to construct a risk signature. Kaplan–Meier survival analysis, enrichment analysis, somatic mutation analysis, immune infiltration analysis, and drug sensitivity analysis were performed to characterize differences between high-risk and low-risk groups. Independent prognostic factors were identified through multivariate Cox regression analysis to construct a nomogram. Expression of signature-related AIRGs was validated using in OC cells and tissues. A total of 109 AIRGs significantly associated with overall survival (OS) in OC were identified, of which 15 were selected to construct the risk signature: AP1S2, AP2A1, ASB2, BTLA, BTN3A3, CALM1, CD3G, CD79A, EVL, FBXO4, FBXO9, HLA-DOB, LILRA2, MALT1, and PIK3CD. This signature stratified the OC cohort into high-risk and low-risk groups, which exhibited significant differences in prognosis, gene expression, mutation profiles, immunotherapy response, and drug sensitivity. Specifically, the low-risk group showed better prognosis, higher tumor mutational burden, greater response to immunotherapy, increased M1 macrophage and T follicular helper (Tfh) cell infiltration, and higher sensitivity to cisplatin and gemcitabine. The nomogram, integrating the AIRG-derived risk signature with age and clinical stage, demonstrated superior performance in predicting OC prognosis compared to other factors. Moreover, the differential expression of signature-related AIRGs were further confirmed in OC cells and tissue as compared to the normal cells or tissues. Our findings highlight the significant association between AIRGs and the prognosis of OC. The prognostic model developed using AIRGs demonstrates strong predictive capabilities.

## 1. Introduction

Ovarian cancer (OC), which is a major threat to women’s health in the United States in 2024 as it is estimated to have 19,680 new cases and 12,740 deaths, due to its high recurrence and mortality rates.^[1]^ Despite advancements in surgical techniques and chemotherapeutic regimens, including the use of platinum-based agents and maintenance therapies with PARP inhibitors and bevacizumab,^[2]^ the 5-year survival rate has seen minimal improvement over the years.^[2]^ Symptoms such as bloating and early satiety can be misleading, often leading to late-stage diagnoses when the cancer has already progressed significantly.^[3]^ Moreover, the recurrence rate is alarmingly high, attributed partly to residual drug-resistant cells and cancer stem cells.^[4]^ Additionally, the interaction between infiltrating immune cells and the ovarian stromal microenvironment through an immunoediting process further complicates the disease’s progression.^[5]^

Recent integrative approaches combining immune checkpoint blockade, PARP inhibition, chemotherapy, and antiangiogenic drugs have shown promise but have not yet achieved substantial breakthroughs in improving survival rates for advanced and metastatic OC patients.^[6]^ With over 300,000 new cases diagnosed annually and approximately 190,000 related deaths expected in 2020 alone,^[7]^ there is a critical need for innovative therapeutic targets and reliable prognostic models to enhance the effectiveness of targeted therapies and to stratify patient prognoses for personalized care. Multigene-based diagnostic models, which have been extensively investigated and applied, demonstrate considerable potential in the prognostic evaluation of cancer patients.^[8,9]^ As technology continues to advance, there is an increasing urgency to develop multigene-based prognostic models specifically for OC to enhance predictive accuracy and personalized medicine approaches.

The adaptive immune system plays a pivotal role in the body’s defense against cancer through mechanisms such as the recognition and elimination of tumor cells.^[10]^ This system involves T-cell receptors and B-cell receptors that can specifically identify and respond to neoantigens presented by cancer cells.^[11]^ However, while this natural surveillance mechanism can induce tumor regression, cancers have evolved strategies to evade immune detection, such as downregulating antigen presentation or upregulating inhibitory ligands like programmed death-ligand 1.^[12]^ Understanding the genetic basis of adaptive immunity could provide insights into why some patients respond better to immunotherapy and how we might predict such responses.^[13]^ Given the significant role of the adaptive immune response in the development and progression of OC, identifying key adaptive immune genes associated with prognosis could lead to the development of novel biomarkers and therapeutic strategies.

**Figure 1.**
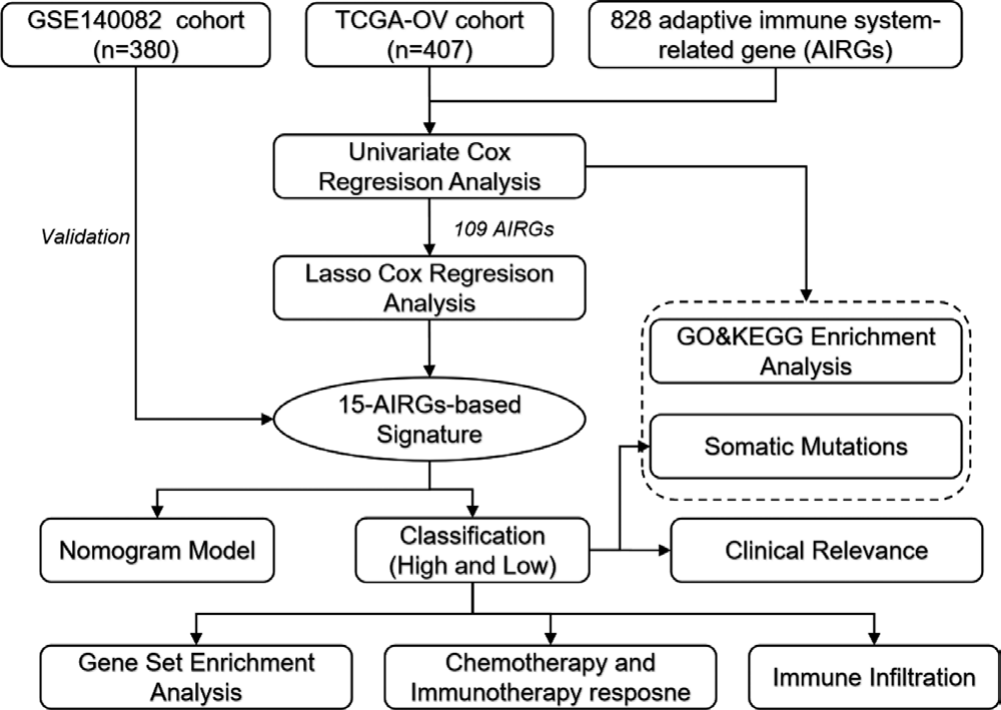
The workflow diagram of the study.

In this study, we utilized cohorts from the cancer genome atlas (TCGA) and the gene expression omnibus (GEO) to systematically analyze the expression, mutation profiles, and prognostic value of adaptive immune-related genes (AIRGs) in OC. Employing machine learning algorithms, we constructed and evaluated a risk signature based on AIRGs for OC. Furthermore, we assessed the utility of this signature in prognostic evaluation and predicting treatment responses in OC patients. Our study focuses on leveraging these insights to construct a robust predictive model based on adaptive immune-related gene expression, aiming to contribute to the growing field of precision medicine in OC. Figure 1 illustrates the workflow of this study.

## 2. Materials and methods

### 2.1. Data acquisition

To establish a predictive model, we obtained transcriptomic, follow-up, and phenotypic data from the TCGA-ovarian serous cystadenocarcinoma (OV) cohort via the University of California Santa Cruz (UCSC) Xena platform (https://xenabrowser.net/datapages/). After excluding cases with incomplete clinical information or follow-up periods shorter than 30 days, a total of 407 OC patient records were included. Additionally, the GSE140082 cohort from the GEO database (https://www.ncbi.nlm.nih.gov/gds/) was selected as an external validation set, consisting of 380 OC samples. The GSE26712 dataset containing gene expression data of 185 OC tissue and 10 normal tissue were downloaded for validating the expression of signature-related AIRGs. A total of 828 AIRGs in the REACTOME_ADAPTIVE_IMMUNE_SYSTEM geneset was sourced from the Molecular Signatures Database (MSigDB, https://www.gsea-msigdb.org/gsea/msigdb/index.jsp) (Table S1, Supplemental Digital Content, http://links.lww.com/MD/O645). Since all data were derived from publicly available databases, no additional ethical approval was required.

### 2.2. Model construction and evaluation

Univariate Cox regression analysis was performed to assess the prognostic relevance of AIRGs. Genes with *P* < .05 were subjected to least absolute shrinkage and selection operator (Lasso) Cox regression analysis using the glmnet package to select feature genes and construct a risk signature. The formula for the risk score was calculated as follows: RiskScore = Σ(βi × mRNAi), where βi represents the coefficient for gene i, and mRNAi denotes the expression level of gene i. Patients were stratified into high-risk and low-risk groups based on the median risk score value. Kaplan–Meier (KM) survival curves and log-rank tests were used to evaluate differences in prognosis between subgroups. Receiver operating characteristic (ROC) curves were utilized to assess the performance of the risk signature.

### 2.3. Enrichment analysis

Differential expression analysis was conducted using the limma package to identify differentially expressed genes (DEGs) in OC. DEGs with *P* < .05 were subjected to enrichment analysis. gene ontology annotation and Kyoto encyclopedia of genes and genomes pathway enrichment analyses were performed using the clusterProfiler package. Additionally, hallmark gene sets were downloaded from the MSigDB database for gene set enrichment analysis (GSEA).

### 2.4. Somatic mutation analysis

Somatic mutations related to single-nucleotide variations in OC patients were analyzed using the maftools package.

### 2.5. Immune landscape analysis

The IOBR package was employed to assess the immune landscape, and the cell-type identification by estimating relative subsets of RNA Transcripts (CIBERSORT) algorithm was used to calculate the infiltration proportions of 22 immune cell types. the estimation of stromal and immune cells in malignant tumor tissues using expression data (ESTIMATE) algorithm was applied to compute stromal scores, immune scores, and tumor purity. Immunophenoscore (IPS) values were calculated using the IPS algorithm.

### 2.6. Treatment response analysis

The tumor immune dysfunction and exclusion (TIDE, http://tide.dfci.harvard.edu/) algorithm was used to evaluate the response of OC patients to immunotherapy. Drug sensitivity was assessed using the pRRophetic package.

### 2.7. Nomogram construction and evaluation

Univariate and multivariate Cox regression analyses were performed to determine whether our signature was an independent prognostic factor. A nomogram was constructed using the “rms” R package. Calibration plots were used to compare the consistency of predicted survival probabilities at 1, 3, and 5 years. Decision curve analysis was employed to evaluate the performance of the nomogram compared to other prognostic factors, and ROC curves were used to assess the predictive accuracy of the nomogram for 1-, 3-, and 5-year survival probabilities.

### 2.8. Validation of the expression of AIRGs

To validate the expression of AIRGs, we retrieved their expression profiles from the Cancer Cell Line Encyclopedia (CCLE, https://sites.broadinstitute.org/ccle/datasets) for both noncancerous and OC cell lines, and generated a heatmap to visualize these expression patterns. Furthermore, we compared the expression levels of AIRGs in OC and normal control tissues in the GSE26712 cohort, to further substantiate the differential expression of AIRGs associated with the risk signature in OC.

### 2.9. Statistical analysis

Statistical analysis and visualization of the bioinformatics data were conducted using R software (version 4.4.1), available at http://www.r-project.org/. Correlation analysis was performed utilizing Pearson correlation coefficient, while inter-group differences were assessed using the Wilcoxon rank-sum test. A *P*-value threshold of < .05 was established to determine statistical significance.

## 3. Results

### 3.1. Expression, mutation, and prognostic characteristics of AIRGs in OC

**Figure 2.**
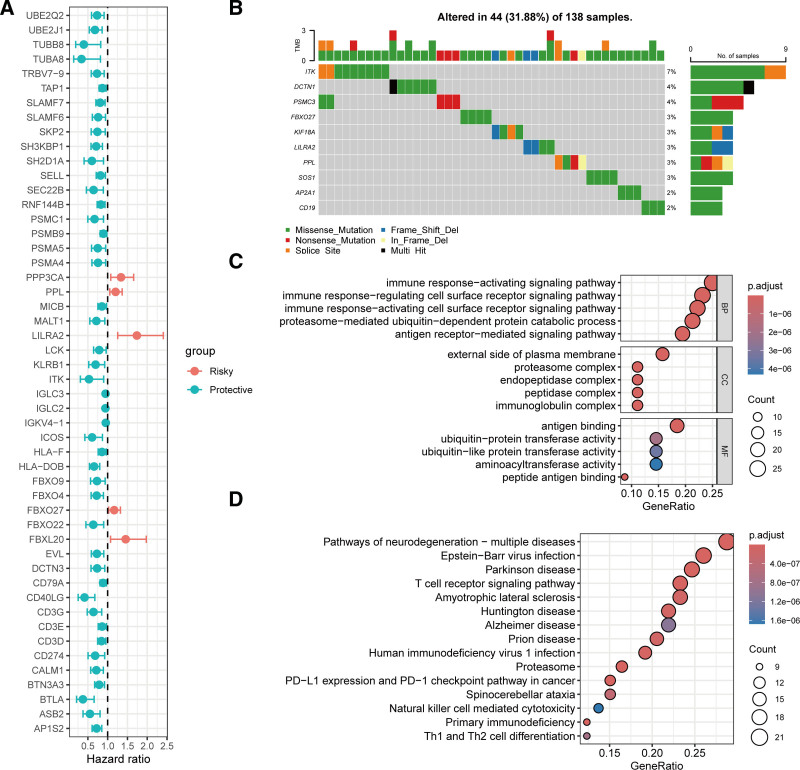
Prognostic relevance of AIRGs in the TCGA-OV cohort. (A) Distribution of hazard ratios for prognostically relevant AIRGs. (B) Oncoplot of the top 10 prognostically relevant genes with the highest mutation frequency. (C) GO enrichment analysis of prognostically relevant AIRGs. (D) KEGG pathway enrichment analysis of prognostically relevant AIRGs. AIRGs = adaptive immune-related genes, GO = gene ontology, KEGG = Kyoto encyclopedia of genes and genomes, OV = ovarian serous cystadenocarcinoma, TCGA = the cancer genome atlas.

To systematically understand the relationship between AIRGs and OC, we analyzed the expression, mutation, and prognostic correlation of AIRGs in the TCGA-OV cohort. Univariate Cox regression analysis identified 109 out of 828 AIRGs significantly associated with overall survival (OS) in OC (Fig. 2A; Table S2, Supplemental Digital Content, http://links.lww.com/MD/O646). Furthermore, among these prognostically relevant AIRGs, the top 10 genes with the highest somatic mutation frequencies are shown in Fig. 2B, with ITK being the most frequently mutated gene. Enrichment analysis revealed that these prognostically relevant AIRGs primarily participate in immune response and ubiquitination processes, exhibiting functions such as antigen binding, ubiquitin transferase activity, and amino acid transaminase activity (Fig. 2C). These genes were also mainly enriched in pathways involving T-cell receptor signaling, natural killer (NK) cell-mediated cytotoxicity, and Th1 and Th2 cell differentiation (Fig. 2D).

### 3.2. Construction of a prognostic signature based on AIRGs

**Figure 3.**
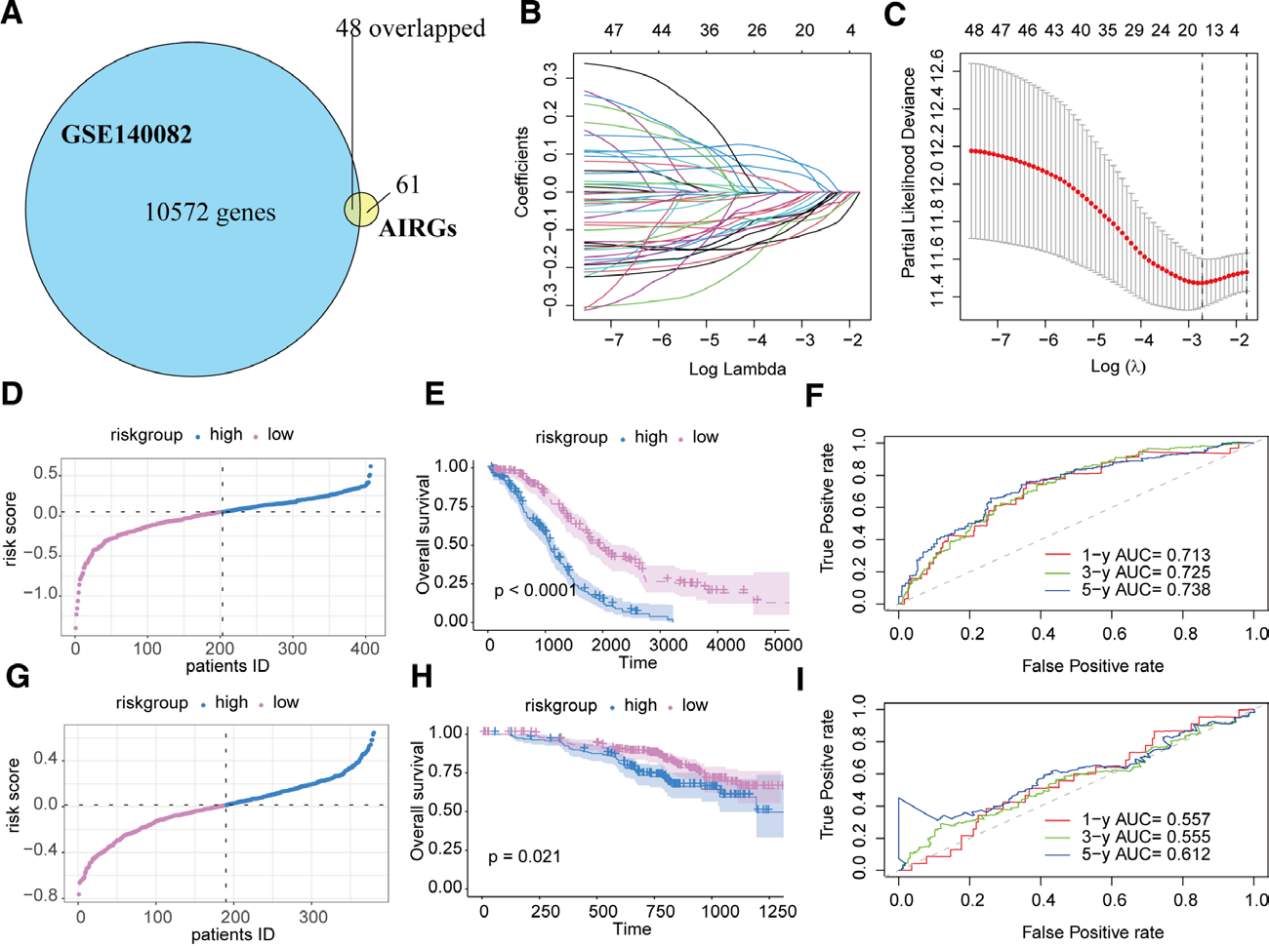
Construction and evaluation of a prognostic signature based on AIRGs in OC. (A) Venn diagram showing the intersection of prognostically relevant AIRGs in the TCGA-OV cohort with the gene set in the GSE140082 cohort. (B and C) Lasso Cox regression analysis for constructing the risk score. (D–F) High-risk and low-risk group stratification, KM survival analysis, and ROC analysis in the TCGA-OV cohort. (G–I) High-risk and low-risk group stratification, KM survival analysis, and ROC analysis in the GSE140082 cohort. AIRGs = adaptive immune-related genes, KM = Kaplan–Meier, OC = ovarian cancer, ROC = receiver operating characteristic, OV = ovarian serous cystadenocarcinoma, TCGA = the cancer genome atlas.

Intersecting the 109 AIRGs significantly associated with OS in OC with the gene set in the GSE140082 cohort resulted in 48 candidate genes for Lasso Cox regression analysis (Fig. 3A). The analysis indicated that selecting 15 genes, including major histocompatibility complex, class II, DO beta (HLA-DOB), adaptor related protein complex 1 subunit sigma 2 (AP1S2), B and T lymphocyte associated (BTLA), leukocyte immunoglobulin like receptor A2 (LILRA2), CD3 gamma subunit of T-cell receptor complex (CD3G), ankyrin repeat and SOCS box containing 2 (ASB2), butyrophilin subfamily 3 member A3 (BTN3A3), F-box protein 4 (FBXO4), calmodulin 1 (CALM1), Enah/Vasp-like (EVL), CD79a molecule (CD79A), MALT1 paracaspase (MALT1), F-box protein 9 (FBXO9), adaptor related protein complex 2 subunit alpha 1 (AP2A1), phosphatidylinositol-4,5-bisphosphate 3-kinase catalytic subunit delta (PIK3CD), minimized the partial likelihood deviance, yielding the best model fit (Fig. 3B and C). The risk score was constructed as follows: −0.104724213 + HLA-DOB – 0.056244202 * AP1S2 – 0.037887773 * BTLA + 0.060571806 * LILRA2 - 0.034947474 * CD3G – 0.043986012 * ASB2 – 0.042773985 * BTN3A3 – 0.085622798 * FBXO4 – 0.071061658 * CALM1 – 0.024210922 * EVL – 0.037338816 * CD79A – 0.005868939 * MALT1 – 0.044942178 * FBXO9 + 0.028006367 * AP2A1 + 0.029351622 * PIK3CD. Using the median risk score, the TCGA-OV and GSE140082 cohorts were divided into high-risk and low-risk groups (Fig. 3D and G). Survival analysis demonstrated that the low-risk group in the TCGA-OV cohort had significantly better OS than the high-risk group (*P* < .0001, Fig. 3E), with the area under the curve (AUC) for predicting 1-, 3-, and 5-year OS being 0.713, 0.723, and 0.738, respectively (Fig. 3F). KM survival analysis showed that the low-risk group in the GSE140082 cohort had significantly better OS (*P* = .021, Fig. 3H), with the AUC for predicting 1-, 3-, and 5-year OS being 0.557, 0.555, and 0.612, respectively (Fig. 3I).

### 3.3. Gene expression profiles across different risk groups

**Figure 4.**
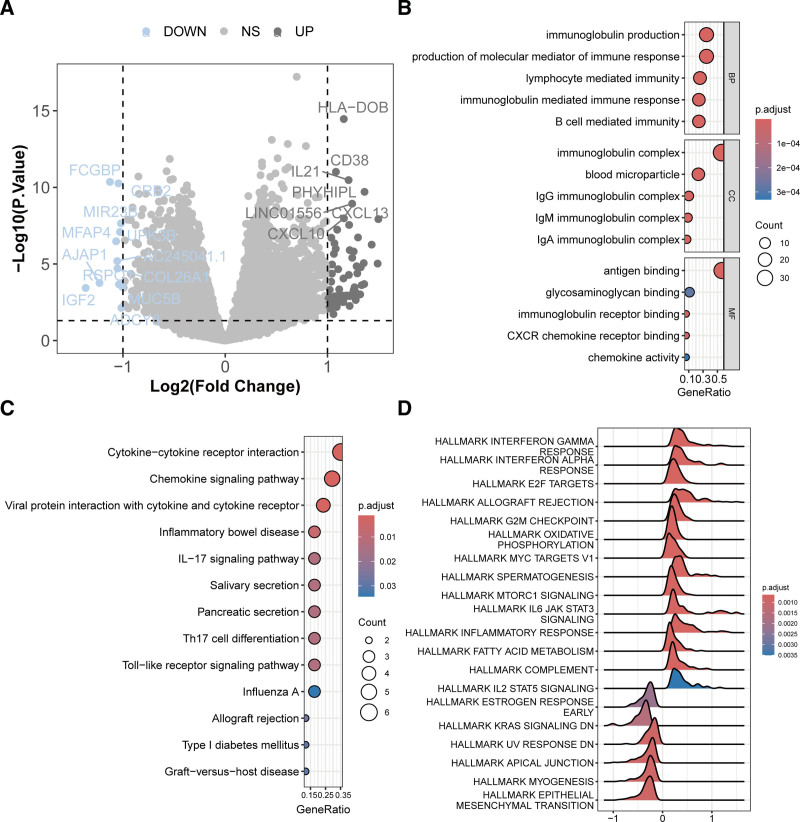
Differential gene expression profiles between high-risk and low-risk groups. (A) Volcano plot of differential expression analysis between high-risk and low-risk groups. (B) GO enrichment analysis of DEGs. (C) KEGG pathway enrichment analysis of DEGs. (D) Hallmark gene set enrichment analysis results. DEG = differentially expressed genes, GO = gene ontology, KEGG = Kyoto encyclopedia of genes and genomes.

To understand the reasons behind the prognostic differences between high-risk and low-risk groups, we analyzed DEGs between the 2 groups. The analysis identified 79 DEGs (*P* < .05, |log2(fold change)| > 1) (Fig. 4A). Enrichment analysis showed that these genes were involved in processes such as immunoglobulin production, immune response, lymphocyte, and B-cell-mediated immune processes (Fig. 4B). Additionally, these genes were mainly enriched in pathways such as cytokine-cytokine receptor interaction, chemokine signaling pathway, IL-17 signaling pathway, Th17 cell differentiation, and Toll-like receptor signaling pathway (Fig. 4C). GSEA indicated that interferon response, E2F targets, allograft rejection, and G2M checkpoint pathways were significantly upregulated in the high-risk group, whereas epithelial mesenchymal transition, myogenesis, and apical junction pathways were significantly downregulated (Fig. 4D).

### 3.4. Mutation profiles across different risk groups

**Figure 5.**
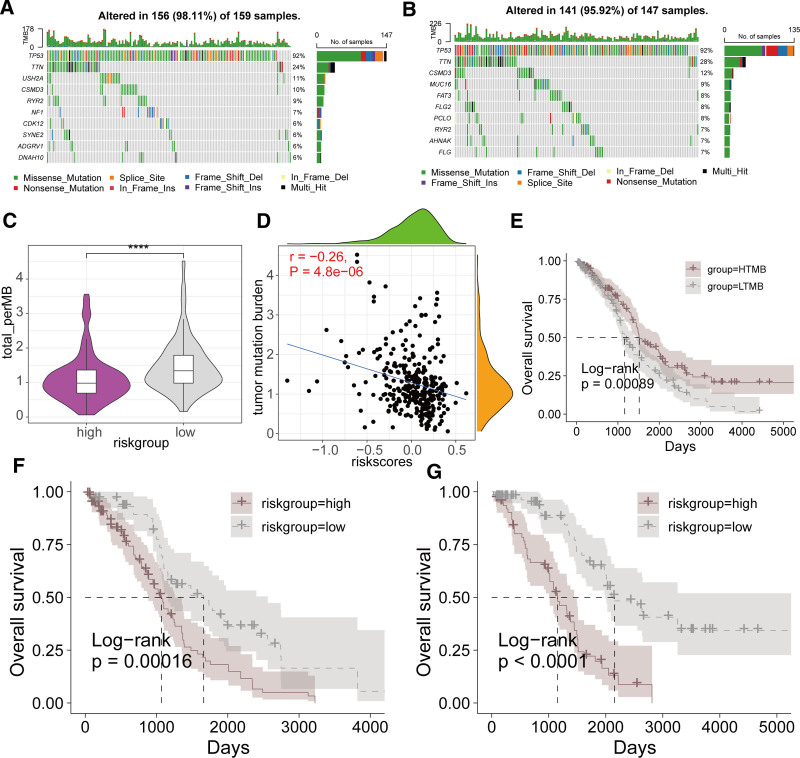
Relationship between the prognostic signature derived from AIRGs and somatic mutations. (A) Mutation landscape of the top 10 most frequently mutated genes in the high-risk group. (B) Mutation landscape of the top 10 most frequently mutated genes in the low-risk group. (C) Comparison of TMB differences between high-risk and low-risk groups. (D) Scatter plot of correlation analysis between TMB and risk score. (E) KM survival analysis curves across different TMB subgroups. (F) KM survival curves between high-risk and low-risk groups in the HTMB subgroup. (G) KM survival curves between high-risk and low-risk groups in the LTMB subgroup. (**** *P* < .0001). AIRG = adaptive immune-related genes, KM = Kaplan–Meier, TMB = tumor mutational burden.

Somatic mutation analysis revealed that tumor protein p53 (TP53) and titin (TTN) were the 2 most frequently mutated genes in both high-risk and low-risk groups, with other genes showing varying mutation frequencies (Fig. 5A and B). Compared to the high-risk group, the low-risk group had higher tumor mutational burden (TMB) (Fig. 5C). There was a significant negative correlation between risk score and TMB (Fig. 5D). Using the median TMB value, we divided the TCGA-OV cohort into high_TMB and low_TMB groups. KM survival analysis indicated that the high_TMB subgroup had significantly better OS than the low_TMB subgroup (*P* = .00089, Fig. 5E). Further analysis of the prognostic differences between high-risk and low-risk groups within high_TMB and low_TMB subgroups showed that high-risk patients had significantly worse OS than low-risk patients (Fig. 5F and G).

### 3.5. Clinical and pathological characteristics across different risk groups

**Figure 6.**
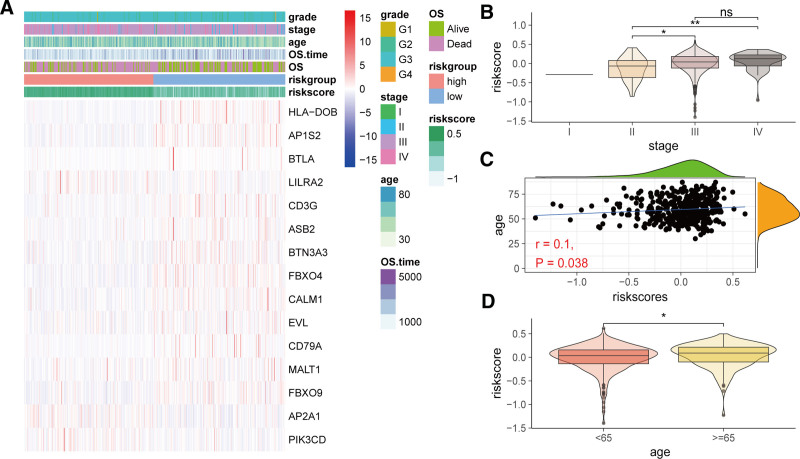
Relationship between the prognostic signature derived from AIRGs and clinical and pathological characteristics. (A) Heatmap of expression of risk signature-related genes and their clinical and pathological characteristics annotations. (B) Comparison of risk scores across different stages. (C) Scatter plot of correlation analysis between age and risk score. (D) Comparison of risk score differences between different age groups. (* *P* < .05, ** *P* < .01). AIRG = adaptive immune-related genes.

We further evaluated the differences in clinical and pathological characteristics between different risk groups. Heatmaps showed that HLA-DOB, AP1S2, BTLA, etc, were highly expressed in the low-risk group, while LILRA2, AP2A1, PIK3CD, etc, were expressed at lower levels. Moreover, the high-risk group contained a higher proportion of older patients and those with advanced stages (Fig. 6A). Therefore, we compared the risk scores across different stages, revealing that advanced stages had higher risk scores than early stages (Fig. 6B). Additionally, age was positively correlated with risk score (Fig. 6C), with the older age group (age ≥ 65) having significantly higher risk scores than the younger age group (age < 65) (Fig. 6D).

### 3.6. Immune landscape across different risk groups

**Figure 7.**
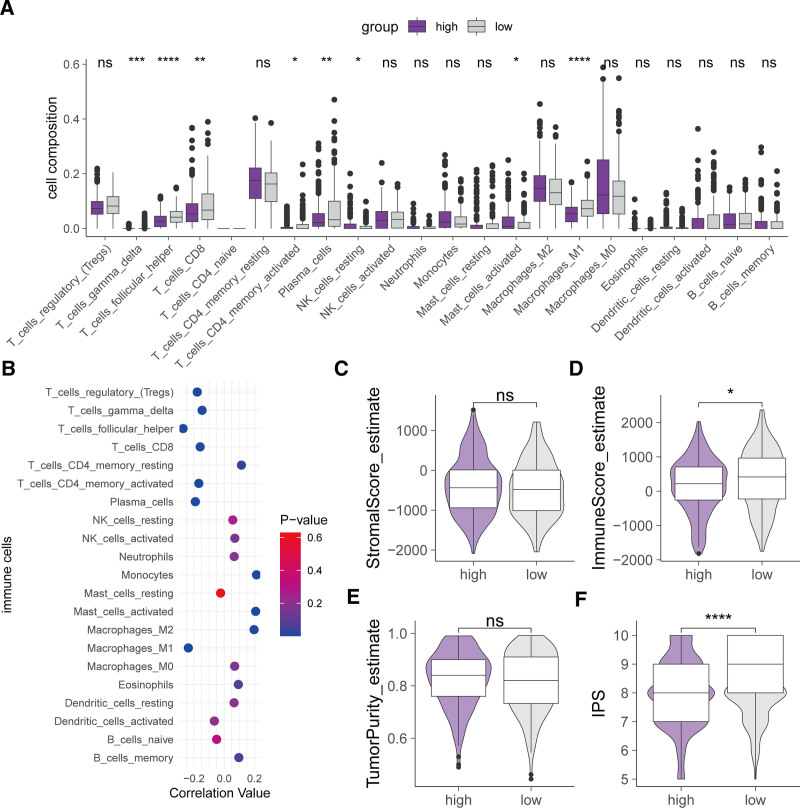
Relationship between risk score and tumor immune microenvironment. (A) Comparison of infiltration proportions of 22 immune cell types between high-risk and low-risk groups. (B) Correlation analysis of immune cell infiltration with risk score. (C) Stromal score, (D) immune score, (E) tumor purity, (F) IPS comparisons between high-risk and low-risk groups. (* *P* < .05, ** *P* < .01, *** *P* < .001, **** *P* < .0001). IPS = immunophenoscore.

Analysis of the tumor immune microenvironment is crucial for prognosis and treatment. Our analysis indicated significant differences in immune cell infiltration between high-risk and low-risk groups. Specifically, the high-risk group had lower infiltration levels of follicular helper T cells, activated CD4 memory T cells, plasma cells, and M1 macrophages compared to the low-risk group, while resting NK cells and activated mast cells were more abundant (Fig. 7A). Correlation analysis showed that the risk score was negatively correlated with the infiltration of most T cells and M1 macrophages but positively correlated with monocytes, mast cells, and M2 macrophages (Fig. 7B). Additionally, compared to the high-risk group, the low-risk group had higher immune scores and IPS, although there were no significant differences in stromal scores and tumor purity (Fig. 7C–F).

### 3.7. Treatment response characteristics across different risk groups

**Figure 8.**
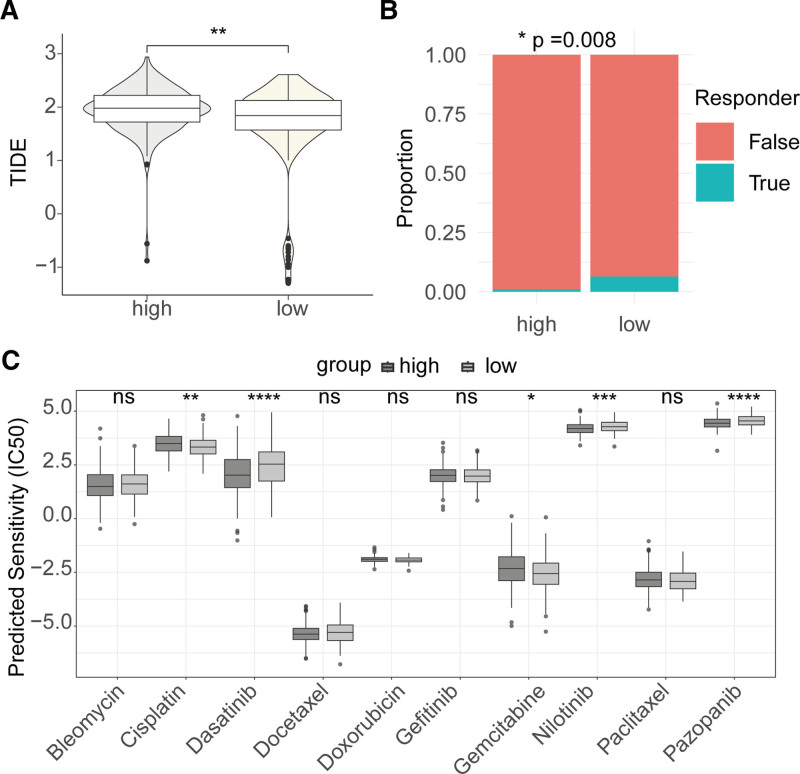
Correlation analysis between risk signature and treatment response. (A) Comparison of TIDE scores between high-risk and low-risk groups. (B) Difference in true and false responders between low-risk and high-risk groups. (C) Comparison of drug sensitivity differences between different risk groups. TIDE = tumor immune dysfunction and exclusion.

To assess the potential value of the AIRG-derived risk signature in treatment response, we first performed TIDE analysis. The low-risk group had lower TIDE scores compared to the high-risk group (Fig. 8A). In the low-risk group receiving immunotherapy, the proportion of true responders was significantly higher than false responders (Fig. 8B). These results suggest that patients in the low-risk group have a higher response rate to immunotherapy. Additionally, we evaluated the sensitivity of different risk groups to ten commonly used drugs, finding that the low-risk group was more sensitive to cisplatin and gemcitabine, while the high-risk group was more sensitive to dasatinib, nilotinib, and pazopanib (Fig. 8C).

### 3.8. Construction of a nomogram for OC

**Figure 9.**
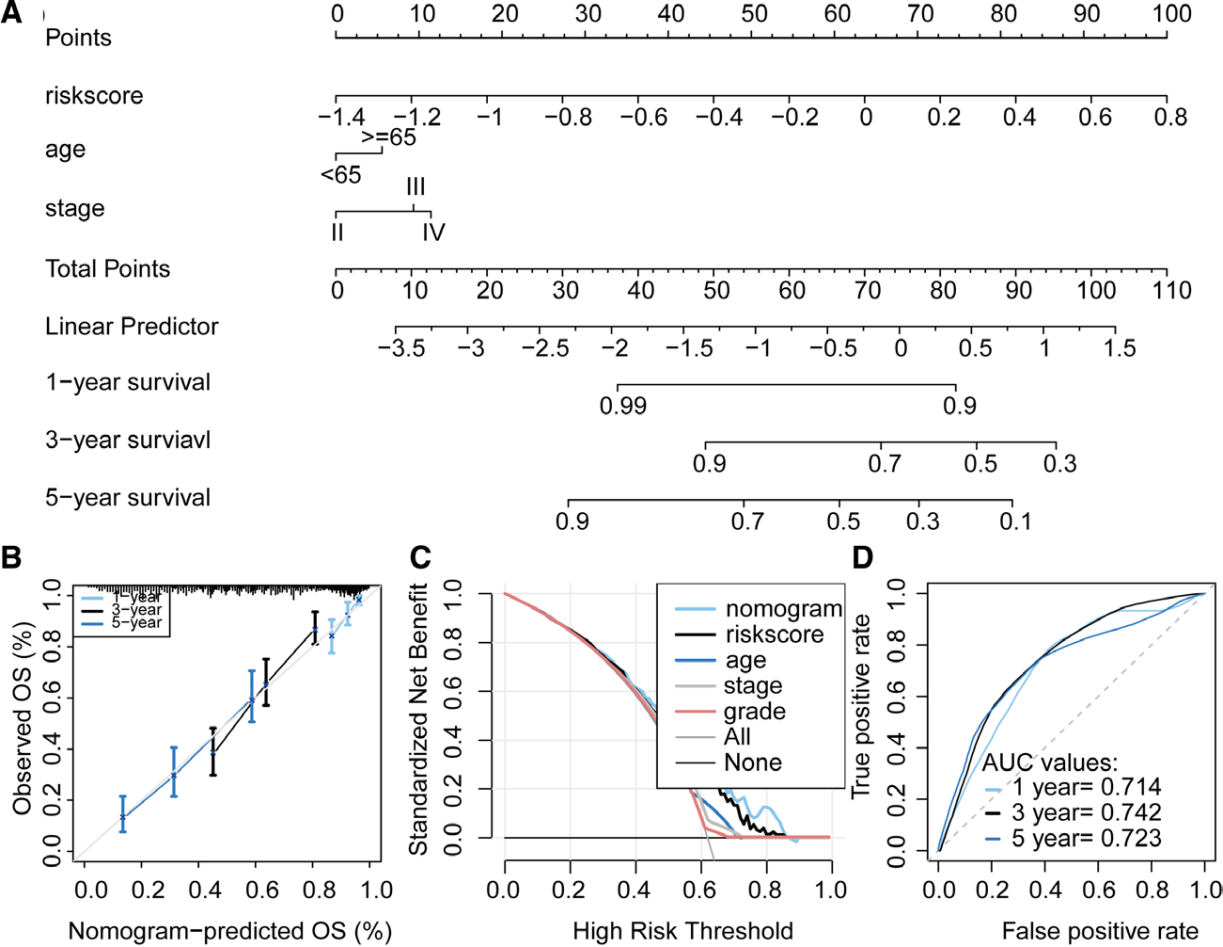
Construction of a nomogram based on the risk signature derived from AIRGs to predict prognosis in OC. (A) Nomogram composed of risk score and stage. Evaluation of nomogram model performance through (B) calibration curve analysis, (C) decision curve analysis, and (D) ROC analysis. AIRG = adaptive immune-related genes, OC = ovarian cancer, ROC = receiver operating characteristic.

We evaluated factors associated with OS in OC and found that the risk score derived from AIRGs, age, and stage were significantly correlated with OS. Multivariate Cox regression analysis confirmed that the risk score and stage were independent prognostic factors for OC (Table 1). Therefore, we constructed a nomogram composed of these 2 factors to predict 1-, 3-, and 5-year OS in OC patients (Fig. 9A). Calibration curves confirmed that the nomogram’s predictions of 1-, 3-, and 5-year OS were closely aligned with actual observations, with narrow error ranges (Fig. 9B). Decision curve analysis showed that the nomogram performed better than other factors in predicting 1-year OS (Fig. 9C). ROC analysis indicated that the nomogram achieved AUC values of 0.714, 0.742, and 0.723 for predicting 1-, 3-, and 5-year OS, respectively (Fig. 9D).

**Table 1:** Factors associated with ovarian cancer prognosis identified by the univariate and multivariate Cox regression analysis.

Characteristics	Univariate		Multivariate	
	HR (95% CI)	*P*-value	HR (95% CI)	*P*-value
Riskscore	4.9 (2.8–8.5)	2.20 × 10^−08^	4.23 (2.41, 7.43)	<.000001
Age	1.4 (1.1–1.8)	.0062	1.41 (1.09, 1.83)	0.009438
Stage	1.5 (1.2–2)	.0025	1.41 (1.08, 1.86)	0.012852
Grade	0.93 (0.65–1.3)	.71	0.90 (0.63, 1.27)	0.535784

HR = hazard ratio.

### 3.9. Validation of the riskscore-related AIRGs in OC cells and tissues

**Figure 10.**
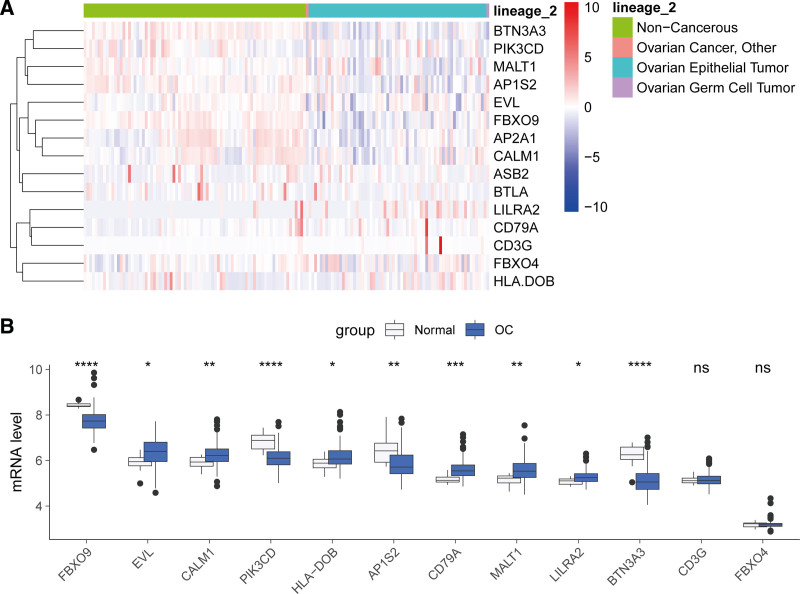
Validation of the expression of riskscore-related AIRGs in OC. (A) The heatmap illustrated the expression of AIRGs in OC and noncancerous cells. (B) Comparison of the expression of AIRGs between the OC and normal tissues in the GSE26712 cohort. AIRG = adaptive immune-related genes, OC = ovarian cancer.

Figure 10A presents a heatmap of the expression profiles for AIRGs associated with a risk score in OC cell lines compared to noncancerous cells from the CCLE. The data reveal that genes such as BTN3A, PIK3CD, MALT1, AP1S2, and FBXO9 exhibit lower expression levels in OC cells, whereas LILRA2, FBXO4, and CD3G show higher expression. Furthermore, by comparing OC tissues with normal tissues in the GSE26712 cohort (Fig. 10B), we observed significant downregulation of FBXO9, PIK3CD, HLA-DOB, AP1S2, BTN3A3, and LILRA2 in OC. Conversely, EVL, CALM1, CD79A, and MALT1 were significantly upregulated in OC. Notably, no significant differences were detected in the expression levels of CD3G and FBXO4 between OC and normal tissues. These findings provide further evidence of the differential expression of AIRGs associated with the risk signature in OC.

## 4. Discussion

The adaptive immune system plays a pivotal role in cancer prevention and control. Cancer typically arises due to genetic mutations within cells leading to uncontrolled proliferation and tumor formation. Multiple cell types and molecular mechanisms within the immune system can recognize and eliminate aberrant cells, preventing cancer progression. This study systematically analyzed the expression, mutation, and prognostic relevance of AIRGs in OC, confirming extensive associations between AIRGs and OC prognosis. Ultimately, a prognostic signature composed of 15 AIRGs was developed, demonstrating potential applications in patient prognosis assessment, tumor immune microenvironment characterization, and treatment response evaluation. A nomogram integrating this risk signature, age, and clinical stage was developed to aid in personalized therapy for OC.

The AIRGs constituting the risk signature have been widely validated in association with cancer. For example, increased expression of BTLA is linked to impaired antitumor immunity and poor outcomes,^[14]^ and preclinical studies have shown that targeting BTLA improves therapeutic efficacy and enhances antitumor immunity.^[15]^ Low BTN3A3 expression correlates with poor prognosis in non-small cell lung cancer,^[16]^ and it inhibits proliferation, metastasis, and invasion of OC cells.^[17]^ CD3G is a novel biomarker for prognosis and immunotherapy in cervical squamous cell carcinoma,^[18]^ and its functional insertion/deletion polymorphisms are associated with hepatocellular carcinoma susceptibility.^[19]^ CD79A expression is associated with the prognosis of oral squamous cell carcinoma and lung cancer.^[20,21]^ EVL is a potential biomarker for pancreatic cancer.^[22,23]^ Ubiquitinating proteins FBXO4 and FBXO9 inhibit tumor metastasis and progression and serve as markers in multiple cancers.^[24,25]^ Previous studies indicate that HLA-DOB and LILRA2 are potential prognostic biomarkers for OC.^[26,27]^ Constitutive activation of MALT1 drives chronic NF-κB target gene expression in hematological malignancies and solid tumors. In cancer, MALT1 activity supports tumor cell survival, proliferation, and metastasis, suggesting that targeting MALT1 may be a promising anticancer strategy.^[28]^ PIK3CD, a catalytic subunit of PI3K, activates downstream effectors like AKT/PKB, influencing cellular growth, survival, metabolism, and playing roles in tumor proliferation, metastasis, and invasion, thus affecting the prognosis of various cancers.^[29,30]^

Immune cell infiltration is critical for tumor growth, progression, and treatment response. Studies show that M1 macrophages exert pro-inflammatory effects, aiding in tumor cell clearance.^[31]^ Higher M1 macrophage infiltration observed in the low-risk group may explain the better prognosis. Tumor-infiltrating lymphocytes (TILs) sometimes form organized tertiary lymphoid structures (TLS), and the presence of functional T follicular helper (Tfh) TILs marks active TLS, correlating with positive clinical outcomes.^[32]^ The prognostic signature derived from AIRGs shows a significant negative correlation with Tfh, with the low-risk score group having higher Tfh cell infiltration, further elucidating the possible mechanism of AIRGs in predicting prognosis.

Tumor immunotherapy has flourished, offering new treatment opportunities for solid tumors. However, responses in OC are often unsatisfactory. Lower-risk signatures correlate with better immunotherapy responses, potentially due to increased Tfh cell infiltration. Studies demonstrate that Tfh mediates checkpoint inhibitor responses in breast cancer mouse models.^[33]^ Additionally, several AIRGs in the risk signature are associated with immunotherapy response. For instance, BTLA is a key checkpoint regulating immune response stimulation and inhibition signals. Its interaction with herpesvirus entry mediator plays a crucial role in negatively regulating immune responses, maintaining immune homeostasis.^[34,35]^ In cancer, abnormal cells exploit checkpoints like BTLA to evade immune surveillance. Thus, high BTLA expression in the low-risk group may influence immune evasion. Cytoplasmic CD79a is a promising biomarker for follow-up after CD19 CAR-T therapy in B-cell leukemia.^[36]^ Additionally, the CD79A/CD40 costimulatory domain endows CAR-T cells with enhanced proliferative capacity and improved antitumor efficacy.^[37]^ Therefore, high CD79A expression in the low-risk group contributes to its immunotherapy response.

This study has some limitations. First, it relies on retrospective datasets from public databases like TCGA, necessitating prospective studies for further validation. Second, the inherent heterogeneity of OC requires larger sample sizes to comprehensively capture its diversity. Lastly, current research lacks real-world validation, including clinical cohorts and animal experiments.

## 5. Conclusion

In summary, this study systematically evaluated the expression, mutation, and prognostic characteristics of AIRGs and successfully constructed a prognostic signature comprised of 15 AIRGs, capable of indicating prognosis, immune landscape, immunotherapy response, and drug sensitivity in OC patients. A nomogram integrating risk score and clinical stage was developed and evaluated for prognostic assessment in OC. Further clinical and basic research is needed to elucidate the roles of AIRGs in OC.

## Author contributions

**Conceptualization:** Huangmin Shi, Lijuan Li, Linying Zhou.

**Data curation:** Huangmin Shi, Lijuan Li, Linying Zhou.

**Formal analysis:** Huangmin Shi, Lijuan Li, Linying Zhou.

**Funding acquisition:** Caiping Hong.

**Investigation:** Huangmin Shi, Lijuan Li, Linying Zhou.

**Methodology:** Huangmin Shi, Lijuan Li, Linying Zhou.

**Project administration:** Caiping Hong.

**Resources:** Huangmin Shi, Lijuan Li, Linying Zhou.

**Software:** Huangmin Shi, Lijuan Li, Linying Zhou.

**Supervision:** Caiping Hong.

**Validation:** Huangmin Shi, Lijuan Li, Linying Zhou.

**Visualization:** Huangmin Shi, Lijuan Li, Linying Zhou.

**Writing – original draft:** Huangmin Shi, Lijuan Li, Linying Zhou.

**Writing – review & editing:** Caiping Hong.

## Supplementary Material


